# The Transmembrane Mucin MUC1 Facilitates β1-Integrin-Mediated Bacterial Invasion

**DOI:** 10.1128/mBio.03491-20

**Published:** 2021-04-06

**Authors:** Xinyue Li, Richard W. Wubbolts, Nancy M. C. Bleumink-Pluym, Jos P. M. van Putten, Karin Strijbis

**Affiliations:** a Department of Biomolecular Health Sciences, Division of Infectious Diseases and Immunology, Faculty of Veterinary Medicine, Utrecht University, Utrecht, Netherlands; University of Illinois at Chicago

**Keywords:** MUC1, transmembrane mucin, bacterial invasion, β1 integrin, ITGB1, host-bacterial interaction, host-bacteria interactions, integrins, mucins

## Abstract

At the intestinal host-microbe interface, the transmembrane mucin MUC1 can function as a physical barrier as well as a receptor for bacteria. MUC1 also influences epithelial cell morphology and receptor function. Various bacterial pathogens can exploit integrins to infect eukaryotic cells. It is yet unclear whether MUC1 influences the interaction of bacteria with integrins. We used Escherichia coli expressing the invasin (inv) protein of Yersinia pseudotuberculosis (E. coli inv) to assess the effects of MUC1 on β1 integrin (ITGB1)-mediated bacterial invasion. Our results show that expression of full-length MUC1 does not yield a physical barrier but slightly enhances E. coli inv uptake. Enzymatic removal of the MUC1 extracellular domain (ED) using a secreted protease of C1 esterase inhibitor (StcE) of pathogenic Escherichia coli had no additional effect on E. coli inv invasion. In contrast, expression of a truncated MUC1 that lacks the cytoplasmic tail (CT) reduced bacterial entry substantially. Substitution of tyrosine residues in the MUC1 CT also reduced bacterial uptake, while deletion of the C-terminal half of the cytoplasmic tail only had a minor effect, pointing to a regulatory role of tyrosine phosphorylation and the N-terminal region of the MUC1 CT in integrin-mediated uptake process. Unexpectedly, StcE removal of the ED in MUC1-ΔCT cells reversed the block in bacterial invasion. Together, these findings indicate that MUC1 can facilitate β1-integrin-mediated bacterial invasion by a concerted action of the large glycosylated extracellular domain and the membrane-juxtaposed cytoplasmic tail region.

## Abstract (precis)

Bacteria can exploit membrane receptor integrins for cellular invasion, either by direct binding of bacterial adhesins or utilizing extracellular matrix components. MUC1 is a large transmembrane glycoprotein expressed by most epithelial cells that can have direct defensive or receptor functions at the host-microbe interface and is involved in facilitating integrin clustering.

## INTRODUCTION

In the healthy human gastrointestinal tract, epithelial cells are separated from the commensal microbiota by a mucus layer, which is the first line of host defense. In the colon, the mucus layer consists mainly of secreted mucins, specific IgA antibodies, and antimicrobial peptides (1). While the outer mucus layer is thick and populated by bacteria, the inner mucus layer is thinner and more difficult to penetrate for bacteria, thus keeping the epithelium and underlying tissue sterile (2). Transmembrane (TM) mucins present on the apical surface of enterocytes are an important component of the inner mucus layer (3). TM mucins have a large *O*-glycosylated extracellular domain (ED) that is linked noncovalently via a SEA (sea-urchin sperm protein, enterokinase, and agrin) module to a transmembrane domain and a cytoplasmic tail (CT) (3). TM mucins form filamentous structures that protrude into the lumen of the gut and are the first point of epithelial contact when pathogens have penetrated the soluble mucus layer (3).

MUC1 is a TM mucin that is expressed by many human epithelial cell types and throughout the gastrointestinal tract (4). The ED of MUC1 extends 200 to 500 nm above the cell membrane, depending on the variable numbers of tandem repeats (VNTR) (5, 6). The ED undergoes extensive *O*-glycosylation, which can vary from about 50% of its molecular mass in the normal mammary gland to about 80% in colon carcinoma cells (7). The CT of MUC1 consists of 72 amino acids with many putative phosphorylation sites (8) and is involved in signaling transduction events.

As a major component of the inner mucus layer, MUC1 plays an important role in interaction with pathogens that can penetrate the mucus layer. Different studies demonstrate that MUC1 forms a defensive barrier that protects against several pathogens. *In vivo* experiments showed that *Muc1* knockout mice have an increased susceptibility to Campylobacter jejuni (C. jejuni) infection and colonization by Helicobacter pylori (H. pylori) (9, 10). Incubation of H. pylori with a MUC1 overexpression cell line showed that MUC1 is shed from the cell surface and that H. pylori was coated with the shed MUC1. These results indicate that MUC1 can act as a decoy receptor in preventing H. pylori adherence to the stomach epithelium (11). In contrast to this barrier function, we recently showed that MUC1 can also act as a receptor for Salmonella enterica subsp. *enterica* serovar Enteritidis (*S.* Enteritidis). Infection assays of confluent HT29-MTX cells with *S.* Enteritidis showed that the *S.* Enteritidis giant adhesin SiiE conferred interaction with MUC1, enabling efficient apical invasion into intestinal epithelial cells (12). The interaction of *S.* Enteritidis SiiE with MUC1 was dependent on sialic acids (12). Others demonstrated that enteroaggregative Escherichia coli (EAEC) colocalized with transiently transfected MUC1 on the surface of HEK293 cells, and this interaction is mediated by binding of its aggregative adherence fimbriae (AAF) to the sialic acids present on MUC1 (13). The binding of AAF with MUC1 also facilitated migration of neutrophils across the epithelium, promoting an inflammatory response to EAEC infection (13).

Membrane rearrangements and endocytosis are essential processes during pathogen invasion. Furthermore, cells bend their membranes into tubulated forms to interact with the environment, including the extracellular matrix (ECM). A recent study showed that MUC1 contributes to the formation of a tubulated membrane morphology and that this is regulated by the mucin size and expression density at the cell surface (14). The tubulated morphology induced by MUC1 was lost after treatment with an enzyme called secreted protease of C1 esterase inhibitor (StcE) (14). StcE is a virulence factor secreted by the human pathogen enterohemorrhagic E. coli (EHEC) O157:H7 (15) that cleaves densely *O*-glycosylated proteins, but not *N*-glycosylated or sparsely *O*-glycosylated substrates (16). StcE cleavage can result in release of the MUC1 extracellular domain from different cells lines (14, 17).

MUC1 affects not only cellular morphology but also cellular behavior. One effect of MUC1 expression is reorganization of integrins, resulting in integrin clustering and promotion of integrin-mediated downstream signaling (18). Integrins are a family of around 24 different α (alpha) β (beta) heterodimeric transmembrane receptors that consist of an extracellular domain, a transmembrane domain, and a cytoplasmic tail (19). Integrins mediate cell-cell, cell-ECM, and cell-pathogen interactions and translate the binding of distinct ligands into intracellular signals that regulate cellular responses (20). Bacterial pathogens can exploit the integrin signaling pathway for adhesion and uptake by nonphagocytic host cells. Integrin-mediated invasion is a common strategy of Yersinia pseudotuberculosis (Y. pseudotuberculosis) and several other bacterial pathogens, including Staphylococcus aureus (S. aureus), *Neisseria* species, and enteroaggregative Escherichia coli (EAEC) (21–25). Various viral pathogens can also utilize integrins for entry (26).

The integrin β1 (ITGB1)-dependent Y. pseudotuberculosis adhesion and entry process is mediated by an adhesion protein called invasin (21). Invasin is a 986-amino acid Y. pseudotuberculosis outer membrane protein encoded by the *inv* gene (21). Multiple members of the ITGB1 family serve as host receptors of invasin (27). Expression of the invasin protein in Escherichia coli (E. coli) is sufficient to promote bacterial invasion into host cells and these E. coli strains have been instrumental in the elucidation of the invasin-integrin invasion pathway (28). So far, MUC1 has been shown to play an important role in direct interaction with different bacteria and in regulating integrin clustering. In this study, we investigated the role of MUC1 in ITGB1-mediated bacterial invasion into host cells.

## RESULTS

### Expression of full-length MUC1 on cells enhances E. coli inv invasion.

**FIG 1:**
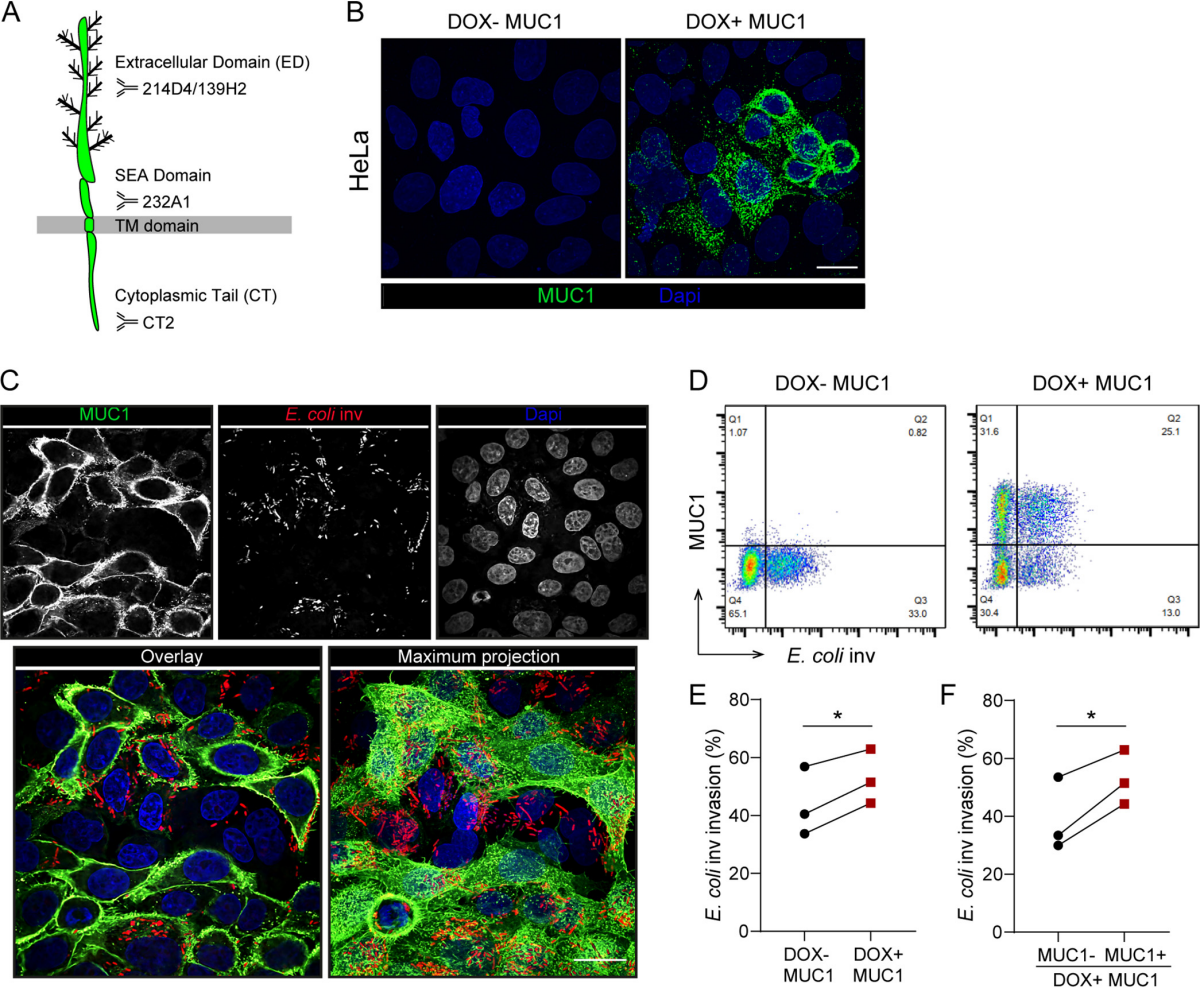
Expression of full-length MUC1 on cells enhances E. coli inv invasion. (A) Schematic model showing that the transmembrane mucin MUC1 contains an extracellular domain (ED), a SEA (sea-urchin sperm protein, enterokinase, and agrin) domain, a transmembrane domain (TD), and a cytoplasmic tail (CT). Recognition sites of antibodies 139H2, 214D4, 232A1, and CT2 are depicted. (B) Immunofluorescence confocal microscopy of HeLa-MUC1 cells induced with 10 μg/ml doxycycline for 24 h and stained with α-MUC1-ED antibody 139H2 (green) and DAPI to stain the nuclei (blue). (C) Immunofluorescence confocal microscopy of DOX+ MUC1 cells induced with 10 μg/ml doxycycline for 24 h, infected with E. coli inv (mCherry, red) at MOI 40 for 2 h and stained with α-MUC1-ED antibody 139H2 (green) and DAPI to stain the nuclei (blue). (D) Flow cytometry of representative DOX− MUC1 cells and DOX+ MUC1 cells infected with E. coli inv (GFP) at MOI 20 for 2 h and stained with α-MUC1-ED antibody 139H2. (E) Percentage of E. coli inv (GFP)-infected cells in DOX− MUC1 cells (Q3/Q3+Q4) and DOX+ MUC1 cells (Q2/Q1+Q2) calculated from (D). (F) Percentage of E. coli inv (GFP)-infected cells in MUC1-positive cells (Q2/Q1+Q2) and MUC1-negative cells (Q3/Q3+Q4) in DOX+ MUC1 cells calculated from (D). Each pair of data points represents the result from a single independent experiment. Three independent experimental replicates are shown. Statistical significance was determined by Student’s *t* test using GraphPad Prism software. *, *P* < 0.05; **, *P* < 0.01; ns, not significant. White scale bars represent 20 μm.

The TM mucin MUC1 is a large glycoprotein that contains three prominent domains. The extracellular domain (ED) has 42 tandem repeats which are heavily *O*-glycosylated and contains an autoproteolytically cleaved SEA domain, a transmembrane domain, and a cytoplasmic tail (CT) that has signaling capacity (29) (Fig. 1A). To investigate the effect of MUC1 on ITGB1-mediated bacterial invasion, we generated a stable HeLa cell line (which lack endogenous MUC1 expression) with a doxycycline-inducible construct encoding full-length MUC1 using lentiviral transduction. After exposure to doxycycline (DOX) for 24 h, MUC1 could be detected on the surface of HeLa cells with the α-MUC1-ED antibody 139H2 that targets a peptide epitope in the tandem repeat region of the extracellular domain. Cells cultured in the absence of DOX did not express any MUC1 (Fig. 1B).

The effect of MUC1 expression on ITGB1-mediated bacterial entry was determined using E. coli strain DH5α expressing the invasin of Yersinia pseudotuberculosis (E. coli inv) together with either a green fluorescent protein (GFP) or fluorescent mCherry protein (30, 31). The invasin protein has previously been shown to confer efficient cellular entry via the ITGB1 receptor (27). Subconfluent DOX− and DOX+ MUC1 cells were incubated with E. coli inv for 2 h. As shown by confocal microscopy, E. coli inv invaded DOX− MUC1 cells as well as DOX+ MUC1 cells (Fig. 1C). We used flow cytometry to quantify MUC1 expression and the total number of infected cells under the two conditions. After incubation with DOX, around 60% of the cells expressed MUC1 (Q1+Q2), while MUC1 expression was not observed in the absence of DOX (Fig. 1D). E. coli inv invasion could be detected under both conditions, specifically that around 35% of the total number of both DOX− and DOX+ MUC1 cells contained E. coli inv (Fig. 1D), suggesting that the induced MUC1 expression did not act as a physical barrier limiting bacterial invasion. Comparison of infection of DOX− MUC1 cells with the MUC1-positive subset of cells in the DOX+ MUC1 cell population yielded 40% infected cells for the MUC1-negative cells (Q3/Q3+Q4) and 50% infected cells for the MUC1-expressing cells (Q2/Q1+Q2) (Fig. 1E). Direct comparison of the percentages of infected MUC1-negative and MUC1-positive subsets of cells within the DOX+ MUC1 cell population similarly showed a small increase in E. coli inv infection for the MUC1-positive cells (Fig. 1F). Together, these results indicate that expression of MUC1 on the cell surface does not form a physical barrier against infection but instead slightly enhances ITGB1-mediated uptake of E. coli inv.

### Enzymatic removal of the extracellular domain of MUC1 does not influence E. coli inv invasion.

**FIG 2:**
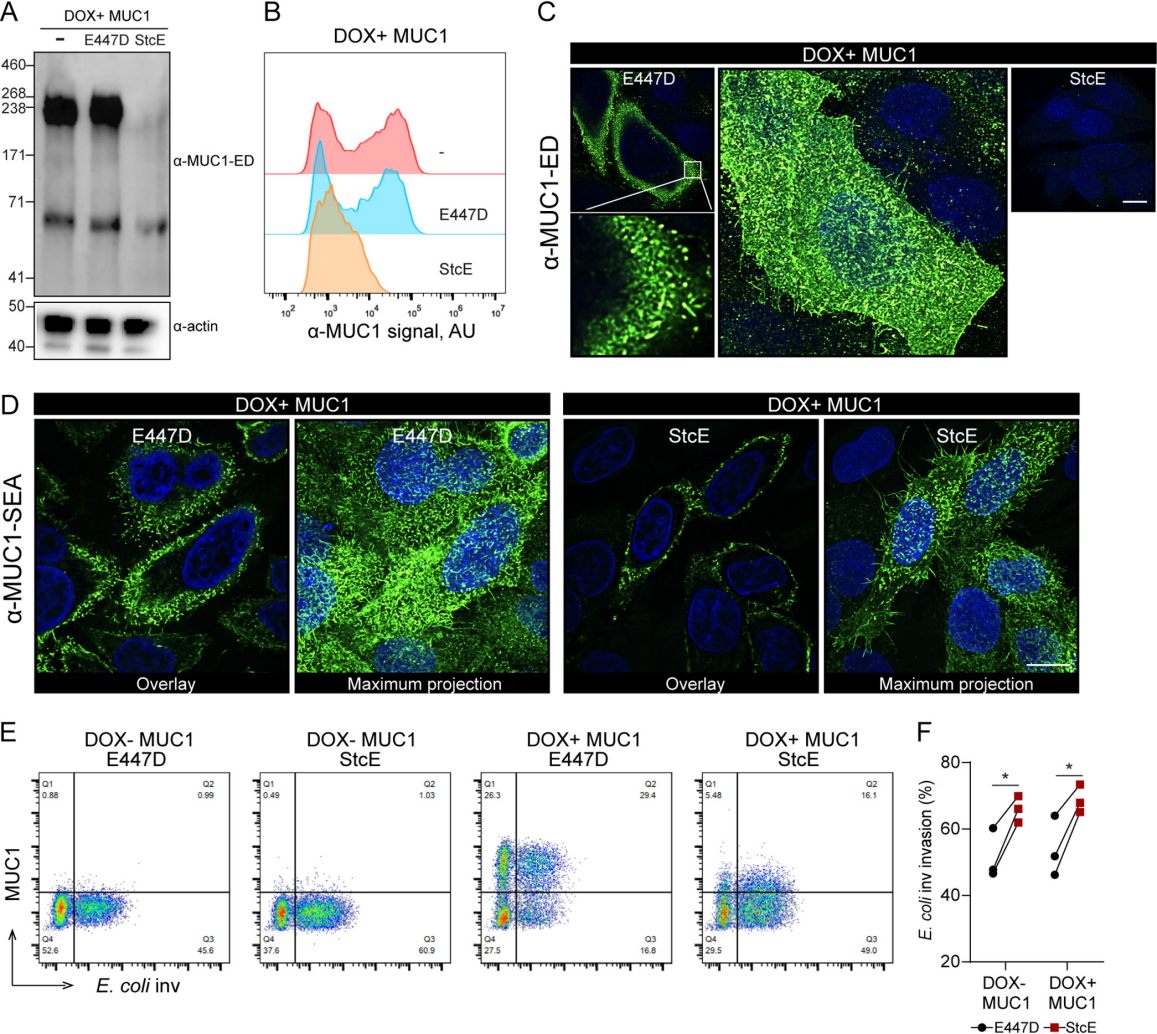
Enzymatic removal of the extracellular domain of MUC1 does not influence E. coli inv invasion. (A) Western blots of DOX+ MUC1 cells treated with 5 μg/ml StcE or E447D for 2 h and stained with α-MUC1-ED antibody 214D4. (B) Flow cytometry of DOX+ MUC1 cells treated with 5 μg/ml StcE or E447D for 2 h and stained with α-MUC1-ED antibody 139H2. (C and D) Immunofluorescence confocal microscopy of DOX+ MUC1 cells treated with 5 μg/ml StcE or E447D for 2 h, stained with α-MUC1-ED antibody 139H2 (green) (C) or α-MUC1-SEA antibody 232A1 (green) (D) and DAPI to stain the nuclei (blue). White scale bars represent 20 μm. (E) Flow cytometry of representative DOX− MUC1 and DOX+ MUC1 cells treated with StcE or E447D, infected with E. coli inv (GFP) at MOI 20 for 2 h, and stained for MUC1 with α-MUC1-ED antibody 139H2. (F) Total infected cell percentage (Q2+Q3) in E447D- or StcE-treated DOX− MUC1 and DOX+ MUC1 cells calculated from (E). Each pair of data points represents the result from a single independent experiment. Three independent experimental replicates are shown. Statistical significance was determined by Student’s *t* test using GraphPad Prism software. *, *P* < 0.05; **, *P* < 0.01; ns, not significant.

The MUC1 ED has been reported to be able to cause reorganization of integrins (18). Therefore, we investigated the role of the ED on E. coli inv invasion by using the bacterial protease StcE to proteolytically remove the highly *O*-glycosylated part of the MUC1 ED from the cell surface (15). We expressed StcE and its mutated inactive form, i.e., the point mutant E447D, as soluble proteins of around 98 kDa, as described previously (32) (Fig. S1A and B in the supplemental material). To verify the effect of StcE on MUC1, DOX+ MUC1 cells were incubated with StcE or E447D for 2 h and then subjected to Western blot analysis with the α-MUC1-ED antibody 214D4. After incubation with StcE, the glycosylated part of the extracellular domain of MUC1 (about 230 kDa) was no longer detectable, whereas the potential intracellular endoplasmic reticulum form of MUC1 (about 70 kDa) was still visible. The inactive enzyme E447D was not capable of removing the 230 kDa band (Fig. 2A). The enzymatic removal of the MUC1 ED by StcE but not E447D was confirmed by flow cytometry analysis (Fig. 2B). Confocal microscopy of the DOX+ MUC1 cells stained with α-MUC1-ED 139H2 showed that MUC1-positive cells displayed hair-like structures, which is in line with the tubulated morphology of MUC1-expressing MCF 10A cells, as was previously described (14). As expected, StcE treatment caused a complete loss of α-MUC1-ED 139H2 staining (Fig. 2C). The MUC1 SEA domain is predicted not to be digested by StcE. Staining of DOX+ MUC1 cells with the α-MUC1-SEA 232A1 antibody confirmed the presence of the hair-like structures in E447D-treated cells and demonstrated a significant reduction of these structures upon treatment with StcE (Fig. 2D). MUC1 SEA domain staining was less intense after StcE treatment and we hypothesize that this could be caused by changes in cell morphology after enzymatic removal of the ED.

To test if the StcE digestion of the MUC1 ED influenced E. coli inv invasion, we determined the total number of bacteria-containing cells in StcE-treated and E447D-treated control cells by fluorescence-activated cell sorting (FACS) analysis. StcE digestion increased the number of bacteria-containing cells in DOX+ MUC1 cells by 1.3-fold. However, this effect was also seen for DOX− MUC1 cells (Fig. 2E and F). These experiments confirmed that the MUC1 ED does not act as a barrier for E. coli inv and suggest that removal of the MUC1 ED has no major impact on the ITGB1-mediated E. coli inv invasion.

### Deletion of the MUC1 cytoplasmic tail reduces E. coli inv invasion.

**FIG 3:**
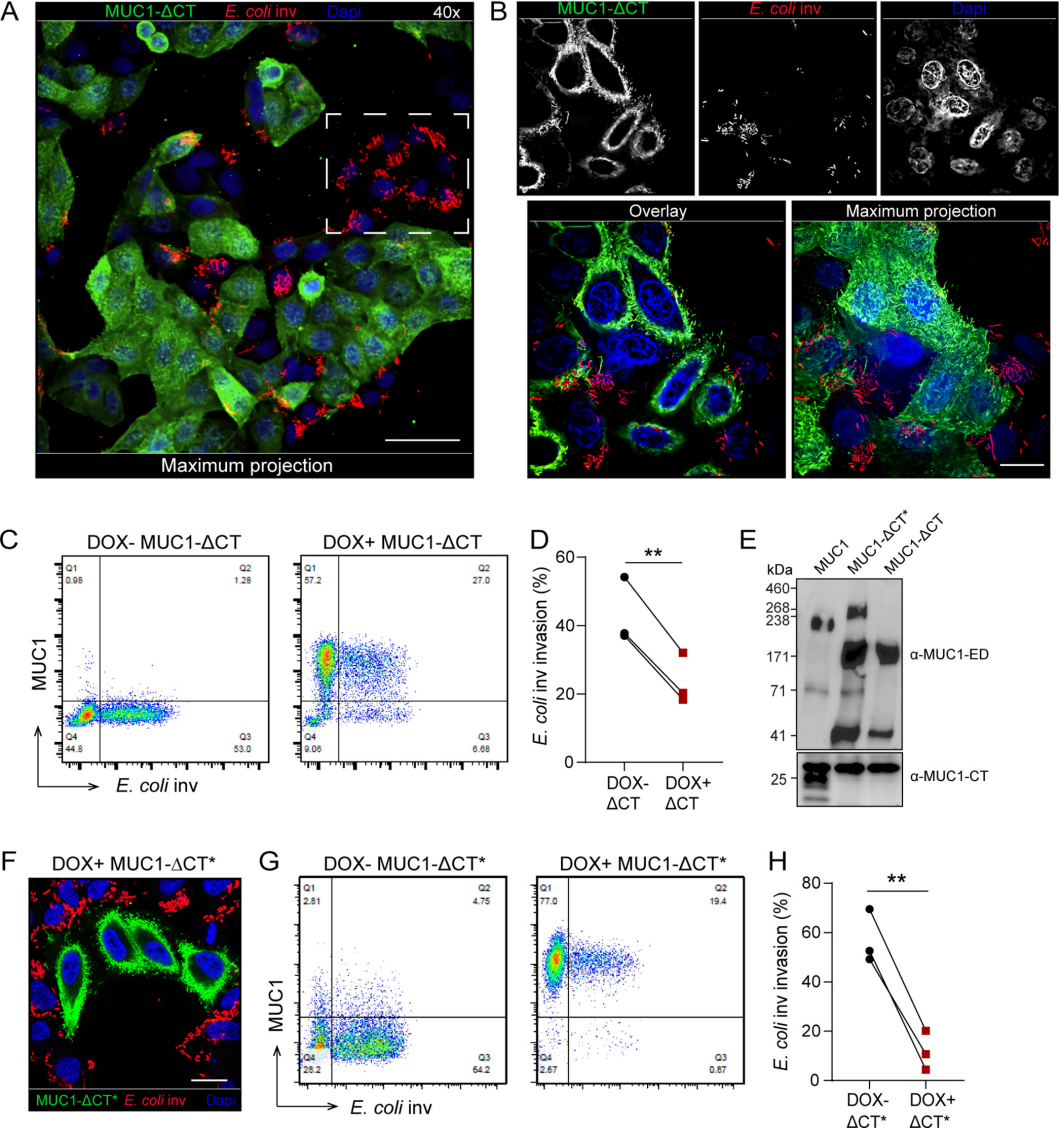
Deletion of the MUC1 cytoplasmic tail reduces E. coli inv invasion. (A and B) Immunofluorescence confocal microscopy of DOX+ ΔCT cells induced with 10 μg/ml doxycycline for 24 h, infected with E. coli inv (mCherry, red) at MOI 40 for 2 h and stained with α-MUC1-ED antibody 139H2 (green) and DAPI to stain the nuclei (blue). White dotted square in (A) denotes infected MUC1-negative cells. Magnification was 40× (A) or 100× (B). (C) Flow cytometry of representative DOX− ΔCT and DOX+ ΔCT cells infected with E. coli inv (GFP) at MOI 20 for 2 h and stained with α-MUC1-ED antibody 139H2. (D) Percentage of E. coli inv (GFP)-infected cells in DOX− ΔCT cells (Q3/Q3+Q4) and DOX+ ΔCT cells (Q2/Q1+Q2) calculated from (C). Each pair of data points represents the result from a single independent experiment. Three independent experimental replicates are shown. Statistical significance was determined by Student’s *t* test using GraphPad Prism software. *, *P* < 0.05; **, *P* < 0.01; ns, not significant. (E) Western blot analysis of DOX+ MUC1, DOX+ MUC1-ΔCT*, and DOX+ MUC1-ΔCT cells detected with α-MUC1-ED antibody 214D4 or α-MUC1-CT antibody F33. (F) Immunofluorescence confocal microscopy of DOX+ ΔCT* cells infected with E. coli inv (mCherry, red) at MOI 40 for 2 h, stained with α-MUC1-ED antibody 139H2 (green) and DAPI to stain the nuclei (blue). (G) Flow cytometry of representative DOX− MUC1-ΔCT* and DOX+ MUC1-ΔCT* cells infected with E. coli inv (GFP) at MOI 20 for 2 h and stained with α-MUC1-ED antibody 139H2. (H) Percentage of E. coli inv (GFP)-infected cells in MUC1-ΔCT* negative cells of DOX− MUC1-ΔCT* cells (Q3/Q3+Q4) and MUC1-ΔCT* positive cells of DOX+ MUC1-ΔCT* cells (Q2/Q1+Q2) calculated from (G). Each pair of data points represents the result from a single independent experiment. Three independent experimental replicates are shown. Statistical significance was determined by Student’s *t* test using GraphPad Prism software. *, *P* < 0.05; **, *P* < 0.01; ns, not significant. White scale bars represent 20 μm.

We next asked whether the cytoplasmic tail of MUC1 could influence ITGB1-mediated E. coli inv invasion. Thus, HeLa cells stably expressing a truncated MUC1 that lacked its cytoplasmic tail (MUC1-ΔCT) were generated. Confocal microscopy showed that these cells still expressed the ED of MUC1 at the cell surface, comparable to the wild-type MUC1 cells (Fig. 3A and B). Microscopy on the DOX+ MUC1-ΔCT cells incubated with E. coli inv showed that cells expressing the MUC1-ΔCT were rarely infected with E. coli, whereas the subset of cells that lacked expression of MUC1-ΔCT showed a high percentage of infection (Fig. 3A and B). Flow cytometry confirmed a significant reduction of about 45% in bacterial uptake by the cells expressing MUC1-ΔCT (Fig. 3C and D).

To validate the results, we tested a second, independently generated clone of MUC1-ΔCT cells, which we named MUC1-ΔCT*. Western blot analysis of these two different clones showed that while the MUC1-ΔCT cells contained two MUC1-specific bands (around 170 kDa and 40 kDa), the MUC1-ΔCT* cells yielded 4 bands (around 230 kDa, 170 kDa, 70 kDa, and 40 kDa) (Fig. 3E). StcE treatment of the MUC1-ΔCT* cells resulted in the disappearance of the upper two bands (230 kDa and 170 kDa), suggesting that both the higher-molecular-weight bands are glycosylated MUC1 isoforms (Fig. S3A). Confocal microscopy of DOX+ MUC1-ΔCT* cells infected with E. coli inv again showed that bacteria were mainly detected in MUC1-ΔCT*-negative cells (Fig. 3F). Flow cytometry analysis indicated a significant (80%) reduction of bacterial uptake by MUC1-ΔCT*-positive cells (Fig. 3G and H). We hypothesize that the larger reduction of E. coli inv invasion in MUC1-ΔCT* compared to MUC1-ΔCT could be linked to the presence of the additional higher-molecular-weight MUC1 isoform in this cell line. The strong inhibition of invasion of E. coli inv in the two independently generated tailless MUC1 cell lines points to a crucial role of the MUC1 cytoplasmic tail on ITGB1-mediated E. coli inv invasion process.

10.1128/mBio.03491-20.3 FIG S3 Removal of the ED of MUC1-ΔCT* cells rescued blocked E. coli inv invasion. (A) Western blot analysis of DOX+ MUC1-ΔCT* cells induced with 10 μg/ml doxycycline for 24 h, treated with 5 μg/ml StcE or E447D for 2 h, and stained with α-MUC1-ED antibody 214D4 or actin. (B) Flow cytometry of representative DOX− MUC1-ΔCT* cells and DOX+ MUC1-ΔCT* cells treated with StcE or E447D, infected with E. coli inv (GFP) at MOI 20 for 2 h, and stained for MUC1 with α-MUC1-ED antibody 139H2. (C) Total infected cell percentage (Q2+Q3) of E447D- or StcE-treated DOX− MUC1-ΔCT* cells and DOX+ MUC1-ΔCT* cells calculated from (B). Each pair of data points represents the result from a single independent experiment. Three independent experimental replicates are shown. Statistical significance was determined by Student’s *t* test using GraphPad Prism software. * *P* < 0.05; ** *P* < 0.01; ns, not significant. (D and E) Western blot analysis of DOX+ MUC1-YF cells and DOX+ MUC1-CT33 cells treated with StcE or E447D and stained with α-MUC1-ED antibody 214D4 or actin. Download FIG S3, TIF file, 2.0 MB. Copyright © 2021 Li et al. 2021 Li et al. https://creativecommons.org/licenses/by/4.0/ This content is distributed under the terms of the Creative Commons Attribution 4.0 International license.

### MUC1 tail attributes are involved in modulating E. coli inv invasion.

**FIG 4:**
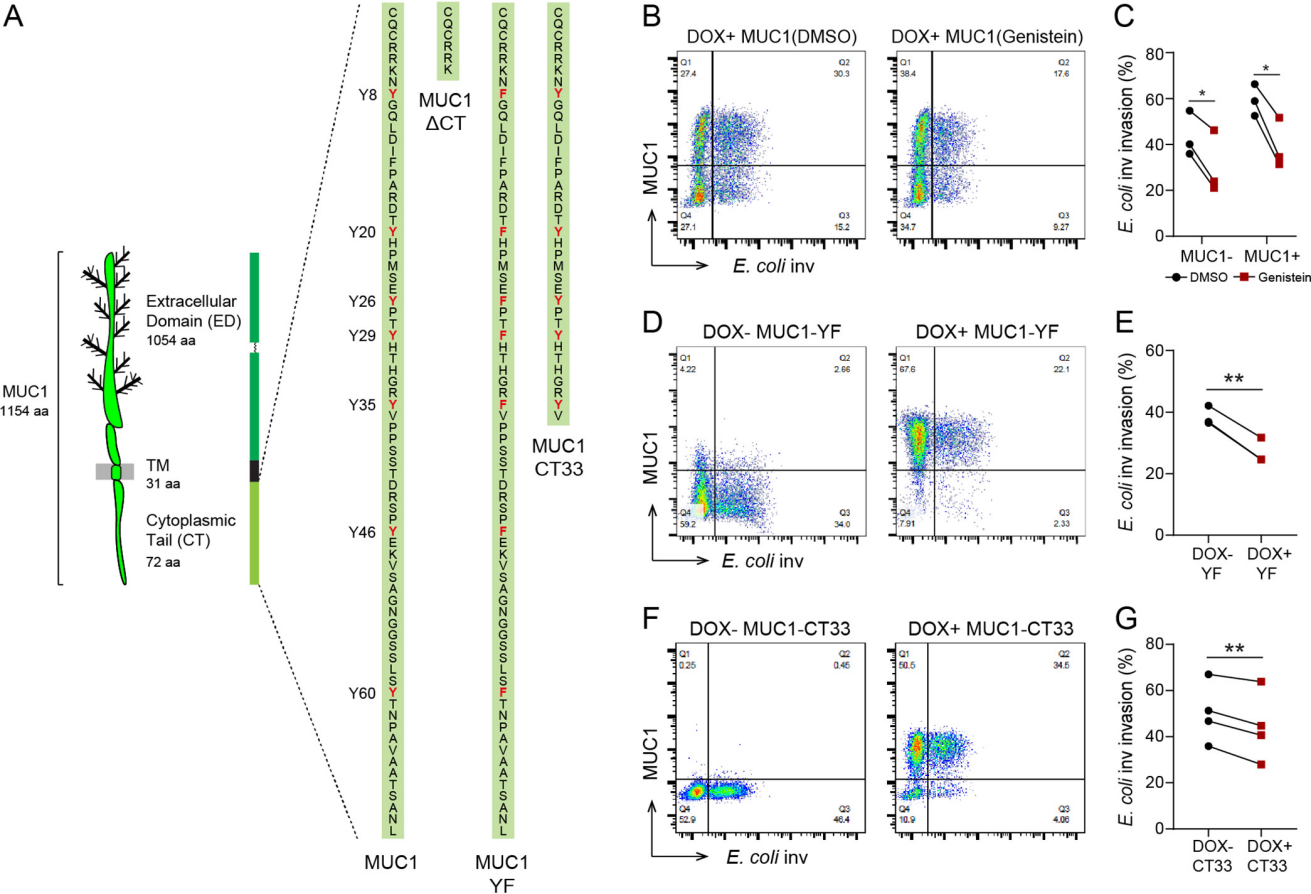
MUC1 tail attributes are involved in modulating E. coli inv invasion. (A) Schematic model showing the domain structure of MUC1 and the protein sequence of the MUC1 cytoplasmic tail and the tails of the MUC1-ΔCT, MUC1-YF, and MUC1-CT33 constructs. Tyrosines (Y) and substituted phenylalanines (F) are indicated in red. (B) Flow cytometry of representative DOX+ MUC1 cells induced with 10 μg/ml doxycycline for 24 h, treated with 75 μM genistein or DMSO for 30 min before infection with E. coli inv (GFP) and for the duration of the infection assay, and stained with α-MUC1-ED antibody 139H2. (C) Percentage of E. coli inv (GFP)-infected cells treated with genistein or DMSO in subpopulation MUC1− or MUC1+ cells of DOX+ MUC1 cells calculated from (B). (D) Flow cytometry of representative DOX− MUC1-YF or DOX+ MUC1-YF cells infected with E. coli inv (GFP) at MOI 20 for 2 h and stained with α-MUC1-ED antibody 139H2. (E) Percentage of E. coli inv (GFP)-infected cells in DOX− MUC1-YF cells (Q3/Q3+Q4) and DOX+ MUC1-YF cells (Q2/Q1+Q2) calculated from (D). (F) Flow cytometry of representative DOX− MUC1-CT33 and DOX+ MUC1-CT33 cells infected with E. coli inv (GFP) at MOI 20 for 2 h and stained with α-MUC1-ED antibody 139H2. (G) Percentage of E. coli inv (GFP)-infected cells in DOX− MUC1-CT33 cells (Q3/Q3+Q4) and DOX+ MUC1-CT33 cells (Q2/Q1+Q2) calculated from (F). Each pair of data points represents the result from a single independent experiment. Three or four independent experimental replicates are shown. Statistical significance was determined by Student’s *t* test using GraphPad Prism software. *, *P* < 0.05; **, *P* < 0.01; ns, not significant.

The MUC1 cytoplasmic tail contains seven putative tyrosine phosphorylation sites (Fig. 4A). To investigate whether these tyrosine residues play a role in regulation of ITGB1-mediated uptake of E. coli inv, we first tested the effect of the general protein tyrosine kinase inhibitor genistein on the bacterial entry process. Incubation of DOX+ MUC1 cells with 75 μM genistein for 30 min prior to bacterial infection and for the entire 2 h infection period reduced the number of E. coli-infected MUC1-positive cells by 34% (Fig. 4B and C). However, this effect could not be attributed to MUC1 specifically, since invasion into MUC1-negative cells was also reduced (30%) (Fig. 4B and C), likely because of the previously described involvement of tyrosine phosphorylation in the ITGB1-mediated uptake process (33).

To specifically investigate the involvement of the tyrosine residues in the MUC1 CT, we substituted all seven tyrosine (Y) residues in the MUC1 cytoplasmic tail to phenylalanines (F) and generated a stable MUC1-YF cell line. These cells expressed MUC1-YF on the cell surface (Fig. S2A). Infection of the DOX+ MUC1-YF cells with E. coli inv showed a 30% reduction in the number of infected cells for the subset of MUC1-YF expressing cells compared to DOX− MUC1-YF cells (Fig. 4D and E), suggesting that tyrosine residues in the MUC1 CT play a role in ITGB1-mediated bacterial uptake.

10.1128/mBio.03491-20.1 FIG S1 Expression of StcE and the inactive point mutant E447D. (A and B) SDS-PAGE gels with different fractions for expression and purification of StcE and E447D. Detection was performed with Coomassie stain. Both StcE and E447D ran at the predicted molecular weight of 98 kDa. PI, pre induction; FT, flow through; E, elution. Download FIG S1, TIF file, 2.6 MB. Copyright © 2021 Li et al. 2021 Li et al. https://creativecommons.org/licenses/by/4.0/ This content is distributed under the terms of the Creative Commons Attribution 4.0 International license.

10.1128/mBio.03491-20.2 FIG S2 Expression and localization of MUC1-YF and MUC1-CT33 in HeLa cells. (A) Immunofluorescence confocal microscopy of HeLa cells expressing MUC1-YF induced with 10 μg/ml doxycycline for 24 h and stained with α-MUC1-ED antibody 139H2 (green) and DAPI to stain the nuclei (blue). (B) Immunofluorescence confocal microscopy of HeLa cells expressing MUC1-CT33 stained with α-MUC1-ED antibody 139H2 (green) and DAPI to stain the nuclei (blue). White scale bars represent 20 μm. Download FIG S2, TIF file, 2.8 MB. Copyright © 2021 Li et al. 2021 Li et al. https://creativecommons.org/licenses/by/4.0/ This content is distributed under the terms of the Creative Commons Attribution 4.0 International license.

To further determine the involvement of different domains in the MUC1 CT on the ITGB1-mediated E. coli inv uptake, we generated a MUC1-CT33 stable cell line that lacks the C-terminal 36 amino acids residues of the CT. DOX induction of the cells confirmed that MUC1-CT33 was expressed on the cell surface (Fig. S2B). Infection assays with these cells demonstrated only a minor decrease (12%) of bacterial uptake by the MUC1-CT33-positive cells compared to MUC1-CT33-negative cells (Fig. 4F and G), which is much less than the strongly reduced uptake observed for the MUC1-ΔCT cells. These data indicate that the N-terminal half of the MUC1 CT is a major factor in modulating ITGB1-mediated E. coli inv invasion.

### Removal of the ED rescues E. coli inv invasion into tailless MUC1 cells.

**FIG 5:**
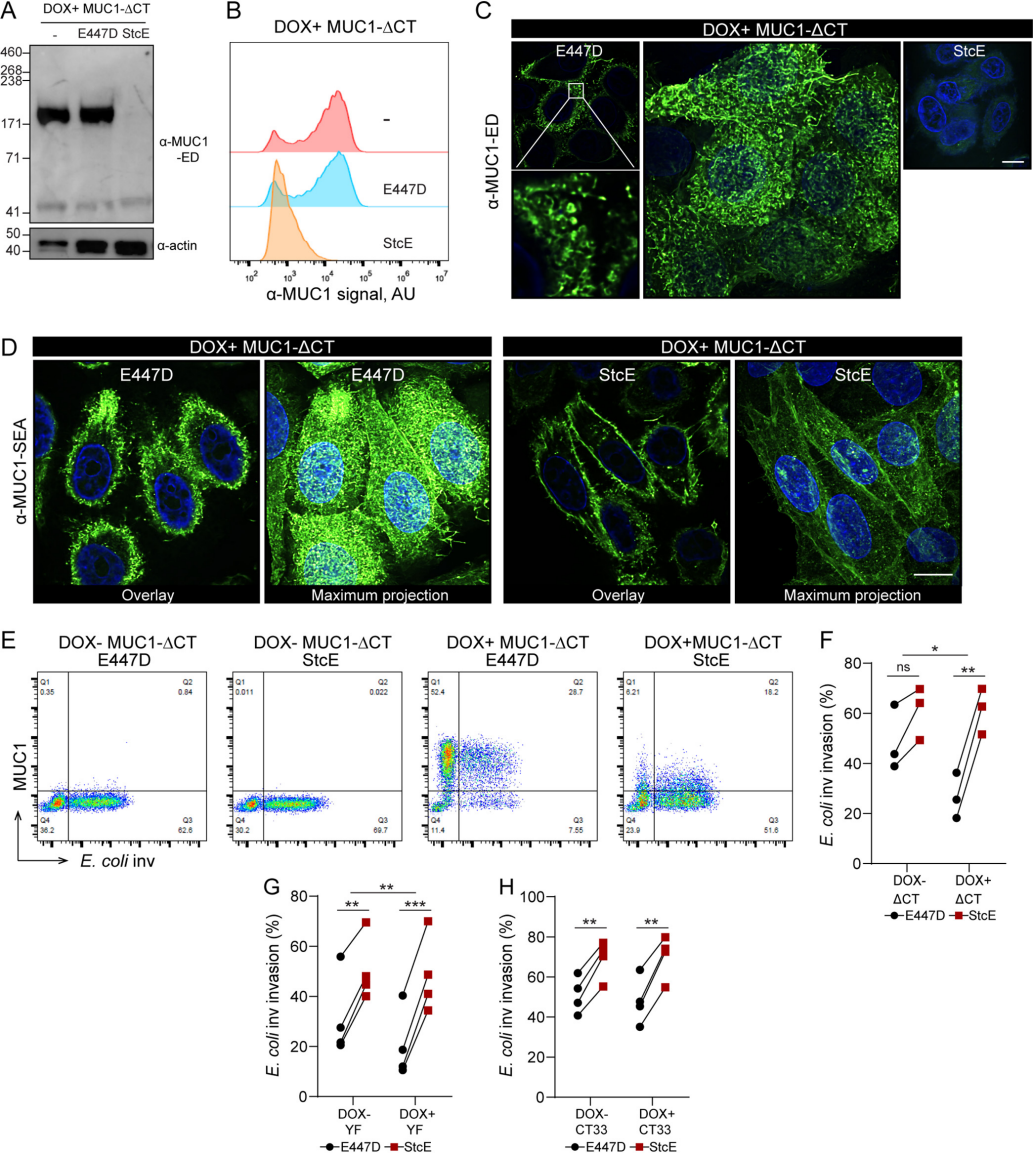
Removal of ED rescues E. coli inv invasion into MUC1-ΔCT cells. (A) Western blot analysis of DOX+ MUC1-ΔCT cells induced with 10 μg/ml doxycycline for 24 h, treated with 5 μg/ml StcE or E447D for 2 h and stained with α-MUC1-ED antibody 214D4 or actin. (B) Flow cytometry of DOX+ MUC1-ΔCT cells treated with 5 μg/ml StcE or E447D for 2 h and stained with α-MUC1-ED antibody 139H2. (C and D) Immunofluorescence confocal microscopy of DOX+ MUC1-ΔCT cells treated with StcE or E447D, stained with α-MUC1-ED antibody 139H2 (green) (C) or α-MUC1-SEA antibody 232A1 (green) (D) and DAPI to stain the nuclei (blue). White scale bars represent 20 μm. (E) Flow cytometry of representative DOX− MUC1-ΔCT cells and DOX+ MUC1-ΔCT cells treated with StcE or E447D, infected with E. coli inv (GFP) at MOI 20 for 2 h, and stained for MUC1 with α-MUC1-ED antibody 139H2. (F) Total infected cell percentage (Q2+Q3) in E447D-or StcE-treated DOX− MUC1-ΔCT cells and DOX+ MUC1-ΔCT cells calculated from (E). Each pair of data points represents the result from a single independent experiment. Three independent experimental replicates are shown. (G) Total infected cell percentage (Q2+Q3) in E447D- or StcE-treated DOX− MUC1-YF cells and DOX+ MUC1-YF cells. Each pair of data points represents the result from a single independent experiment. Four independent experimental replicates are shown. (H) Total infected cell percentage (Q2+Q3) in E447D- or StcE-treated DOX− MUC1-CT33 cells and DOX+ MUC1-CT33 cells. Each pair of data points represents the result from a single independent experiment. Four independent experimental replicates are shown. Statistical significance was determined by Student’s *t* test using GraphPad Prism software. *, *P* < 0.05; **, *P* < 0.01; ns, not significant.

The ED of MUC1 has been reported to influence integrin activities by reorganizing integrins and funneling active integrins into focal adhesions (18). Removal of the ED of MUC1 altered the curved plasma membrane morphology (14). To further investigate the strong reduction in ITGB1-mediated bacterial uptake by the MUC1-ΔCT cells, we treated these cells with StcE to proteolytically remove the MUC1 ED. After StcE treatment, the ED of the MUC1-ΔCT cells was no longer detectable as determined by Western blotting and flow cytometry (Fig. 5A and B). Similar to cells expressing MUC1, the MUC1-ΔCT cells also displayed hair-like membrane structures, as detected by confocal microscopy with the α-MUC1-ED 139H2 and α-MUC1-SEA 232A1 antibodies (Fig. 5C and D). StcE treatment of the MUC1-ΔCT cells resulted in a loss of α-MUC1-ED 139H2 staining (Fig. 5C) and staining of the unaffected SEA domain demonstrated a clear loss of the prominent hair-like membrane features (Fig. 5D). Infection assays showed that StcE treatment of MUC1-ΔCT caused a 2.3-fold increase in the number of E. coli inv-infected DOX+ MUC1-ΔCT cells compared to 1.2-fold for the DOX− MUC1-ΔCT cells (Fig. 5E and F). In the DOX+ MUC1-ΔCT* cells, the rescue of the MUC1-ΔCT* defect in bacterial uptake by StcE treatment was even more pronounced, with a 5-fold increase in the number of infected cells compared to 1.2-fold for the DOX− MUC1-ΔCT* cells (Fig. S3B and C). The rescue of bacterial uptake in both MUC1-ΔCT and MUC1-ΔCT* cells by StcE treatment, to levels comparable to MUC1-negative cells, points to a barrier or inhibitory role of the ED in the absence of the CT.

To better understand the StcE-mediated rescue of ITGB1-mediated bacterial invasion in the MUC1-ΔCT cells, we assessed the effect of StcE removal of the ED in MUC1-YF and MUC1-CT33 cells on E. coli inv invasion. Western blot analysis showed that the EDs of DOX+ MUC1-YF and DOX+ MUC1-CT33 cells were no longer detectable after StcE treatment (Fig. S3D and E). Infection assays showed that StcE treatment increased bacterial invasion in the DOX+ MUC1-YF cells by 2.4-fold, compared to 1.6-fold for the DOX− MUC1-YF cells, resulting in comparable infection percentages in the two conditions (Fig. 5G). In MUC1-CT33 cells, StcE treatment yielded a comparable 1.4-fold increase in both DOX− and DOX+ cells (Fig. 5H), as was observed for the cells expressing MUC1 (Fig. 2E and F). These results indicate that the ED of MUC1 plays an essential role in the strong reduction of the bacterial invasion in the tailless MUC1 cells and, moreover, that a cooperative action of the ED and the N-terminal domain of the CT of MUC1 is needed for modulation of ITGB1-mediated bacterial invasion.

## DISCUSSION

At the interface between microbiota and the intestinal epithelium, the TM mucin MUC1 plays different roles in preventing and facilitating bacterial invasion. The ITGB1 receptor is exploited by different pathogens, including Yersinia pseudotuberculosis, Staphylococcus aureus, *Neisseria* species, and enteroaggregative Escherichia coli (EAEC), to adhere to or invade mammalian cells (21–25). Our study demonstrates for the first time that MUC1 can facilitate ITGB1-mediated bacterial invasion. We present evidence that the extracellular domain and cytoplasmic tail of MUC1 collectively control this process. Using E. coli inv ITGB1-mediated bacterial invasion as a model system, we unequivocally demonstrate that MUC1 has no barrier function against E. coli inv and that removal of the MUC1 ED barely influences the E. coli inv uptake process (Fig. 1, Fig. 2). On the contrary, deletion of the CT of MUC1 strongly reduced bacterial invasion (Fig. 3). Substitution of tyrosines to phenylalanines in the MUC1 cytoplasmic tail reduced bacterial uptake, and deletion of the C-terminal half of the CT only had a minor effect of bacterial uptake, pointing to a role of tyrosine phosphorylation and the N-terminal region of the CT in influencing bacterial entry (Fig. 4). Remarkably, enzymatic removal of the ED in MUC1-ΔCT cells substantially reversed the defects in ITGB1-mediated invasion (Fig. 5). Together, these data indicate that the ED and CT of MUC1 act jointly in modulating ITGB1-mediated bacterial entry.

Conventionally, MUC1 is considered a defense barrier protecting the underlying epithelium. On the other hand, pathogens may exploit MUC1 as a receptor. For example, we recently showed that MUC1 is a receptor for *Salmonella* apical invasion into enterocytes (12). Our present results indicate yet another scenario, namely that MUC1 influences the ITGB1-mediated invasion of bacteria. The prominent role of MUC1 in E. coli inv invasion was unexpected, as E. coli inv has not been reported to interact with MUC1 directly and MUC1 overexpression in breast cancer cells prevents ITGB1-mediated cell adhesion to extracellular matrix components (34). Furthermore, a recent study has demonstrated that the MUC1 ED is able to reorganize cell surface receptor integrins (18). This MUC1-induced reorganization primes integrin clustering and funnels active integrins into focal adhesions, thus altering integrin activation status and their capacity to interact with their ligands (18). It can be imagined that such a process would result in increased ITGB1-mediated bacterial uptake. However, in our experiments, enzymatic removal of the ED of MUC1 using the enzyme StcE showed only a slight increase in bacterial entry that was not dependent on the presence of MUC1 (Fig. 2). Therefore, the MUC1 ED may not be crucial in regulating the ITGB1-mediated bacterial invasion when the MUC1 cytoplasmic tail is present.

In contrast to the negligible effect of removal of the MUC1 ED, deletion of the CT of MUC1 caused a pronounced reduction of ITGB1-mediated bacterial invasion (Fig. 3). The cytoplasmic tail of MUC1 consists of 72 amino acids, among which are multiple residues that can be phosphorylated (8). Although MUC1 has been one of the most intensively studied mucins, signaling through the MUC1 tail is not well understood. One clear link between bacterial stimulation and MUC1 cytoplasmic tail signaling was demonstrated for the airway pathogen Pseudomonas aeruginosa. Binding of this bacterium or its flagellin protein to hamster Muc1 expressed in Chinese hamster ovary (CHO) cells stimulates phosphorylation of the Muc1 CT and activates the MAP kinase pathway (35–37). Besides this direct signaling through MUC1 CT, not much evidence has been provided that MUC1 CT plays a role in regulating receptor-mediated bacterial adhesion or invasion. Signaling of ITGB1 has been studied in detail and engagement by E. coli inv enables ITGB1 to act as a traditional receptor in transmitting an “outside-in” signaling. Many proteins like tyrosine kinases, including focal adhesion kinase (FAK), Src, paxillin, phosphoinositide 3-kinase (PI3K), and Rho family member Rac1, have been shown to play important roles in ITGB1-mediated bacterial uptake (38–40). The tail of MUC1 contains 7 tyrosine residues, of which 6 are 100% conserved across mammalian species (41). We hypothesized that these tyrosines could play a role in the regulation of tyrosine kinases downstream of ITGB1. Expression of a MUC1 construct in which all tyrosines in the cytoplasmic tail were replaced by phenylalanines (MUC1-YF) indeed reduced E. coli inv entry (Fig. 4), suggesting that tyrosine phosphorylation in the MUC1 CT contributes to the process of bacterial uptake. The finding that truncation of the C-terminal half of the CT only had a minor effect on bacterial uptake indicated that this part of the tail is not essential. Therefore, we conclude that the N-terminal 36 amino acids of the CT proximal to the membrane play a major role in regulating the process of bacterial uptake through ITGB1 (Fig. 4).

The importance of the CT domain during modification of the ITGB1 receptor activity was confirmed with two independently generated MUC1-ΔCT cell lines (MUC-ΔCT and MUC-ΔCT*). Both cell lines showed a markedly reduced uptake of E. coli inv, with the most pronounced effect observed for the MUC1-ΔCT* cells (Fig. 3). Notably, these cells exhibited an additional high molecular weight band by Western blot probed for the MUC1 ED. Both upper bands disappeared after StcE treatment, suggesting that both are MUC1 *O*-glycosylated EDs. Based on these results, we hypothesize that MUC-ΔCT and MUC-ΔCT* may represent different MUC1 isoforms, as has been observed in other studies (42). StcE treatment of the ED in both MUC1-ΔCT and MUC1-ΔCT* cells reversed the blocked bacterial uptake, suggesting that the ED functions as a barrier in tailless MUC1, probably by changing membrane morphology and reorganizing ITGB1. The presence of a larger ED in MUC1-ΔCT* cells could contribute to the stronger reduction of bacterial uptake compared to the MUC1-ΔCT cells.

**FIG 6:**
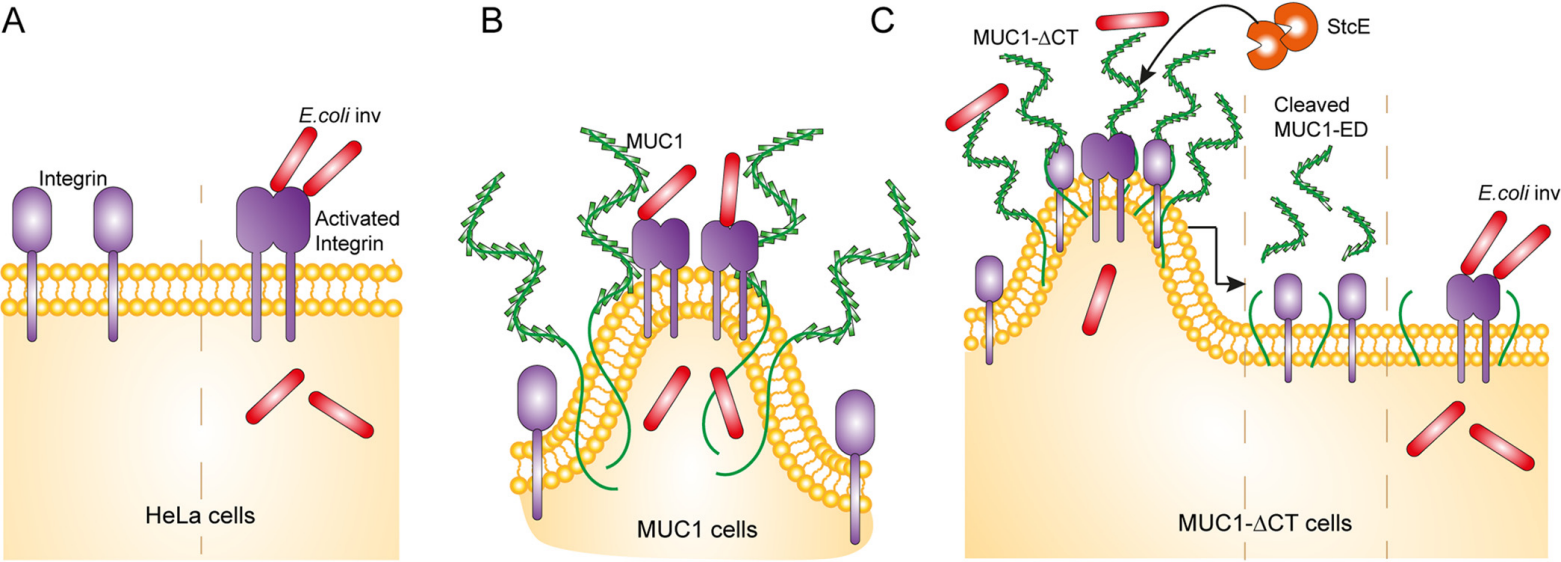
Working model describing the impact of MUC1 on ITGB1-mediated E. coli inv invasion. (A) In HeLa cells, interaction of E. coli inv with ITGB1 initiates uptake of bacteria. (B) Working model of E. coli inv interaction with ITGB1 in HeLa cells expressing MUC1. MUC1 does not form a barrier that prevents bacterial invasion. The MUC1 extracellular domain alters the cell membrane to tubulated morphology and reorganizes ITGB1, which may reduce overall integrin-bacteria binding, but primes integrin clustering. In this way, expression of MUC1 promotes bacterial uptake instead of being a barrier. (C) Working model of E. coli inv interaction with ITGB1 in HeLa cells expressing MUC1 without the cytoplasmic tail (MUC1-ΔCT). The presence of the MUC1 ED alters the cell membrane to tubulated morphology and reorganizes ITGB1, which may reduce overall integrin-bacteria binding. In the absence of a functional CT, priming and stabilization of integrin clusters will not occur, and this leads to severely reduced bacterial uptake. StcE treatment of MUC1-ΔCT cells removes the ED and reduces the tubulated morphology of the membrane, which leads to recovery of overall integrin-bacteria binding, ITGB1 outside-in signaling, and restored bacterial uptake.

Our findings that expression of MUC1-ΔCT strongly reduced ITGB1-mediated bacterial invasion and that StcE treatment of ED in MUC1-ΔCT cells rescued this effect suggest that the ED and CT of MUC1 play a joint role in the regulation of ITGB1 activity and E. coli inv invasion. Based on our findings, we consider a working model to explain how the ED and CT of MUC1 impact ITGB1-mediated bacterial invasion (Fig. 6). In the absence of MUC1, E. coli inv engages ITGB1 to invade cells (Fig. 6A). In MUC1 cells, the MUC1 ED does not form a barrier for E. coli inv. Instead, it alters the cell membrane into a tubulated morphology and potentially reorganizes ITGB1 (18). These changes perhaps reduce the overall bacteria-integrin binding, but prime integrin clustering and promote bacterial uptake (Fig. 6B). In MUC1-ΔCT cells, the presence of MUC1 ED could still reorganize ITGB1 and confer cells with tubulated morphology, but E. coli inv invasion is severely reduced. This points to a potential role of the CT in priming and stabilizing integrin clusters. Removal of the MUC1 ED and the coinciding reduction of membrane curvature rescues bacteria-integrin binding and outside-in signaling that allows invasion (Fig. 6C).

Targeting integrins to confer adherence to or invasion into epithelial cells is a common strategy in bacterial pathogenesis. Our finding that MUC1 expression influences ITGB1-mediated bacterial invasion provides more insight into this process. While the Y. pseudotuberculosis invasin binds directly to ITGB1 to invade epithelial cells, several other bacterial species utilize extracellular matrix proteins such as fibronectin, a natural ligand of ITGB1, to connect to epithelial cells. For example, EAEC makes use of fibronectin-mediated binding to α5β1 integrin to adhere to intestinal cells (25). Interestingly, EAEC can also directly engage MUC1 as a host cell receptor (13). This is a unique example in which a pathogen can interact with both MUC1 and integrins to mediate adherence to epithelial cells. It is tempting to assume there may be a link between human MUC1 and ITGB1 to mediate EAEC adherence and to determine the course of diseases.

In conclusion, we demonstrate for the first time that the TM mucin MUC1 could modulate ITGB1-mediated bacteria invasion and that the extracellular domain and cytoplasmic tail of MUC1 act cooperatively in this process. The modulation of ITGB1 by MUC1 underscores the significance of TM mucins in the pathogenesis of various pathogens.

## MATERIALS AND METHODS

### Cell culture.

HeLa cells and HEK293T cells were routinely grown in 25 cm^2^ flasks in Dulbecco’s modified Eagle’s medium (DMEM) containing 10% fetal calf serum (FCS) at 37°C in 5% CO_2_. For use in confocal microscopy, cells were cultured on circular glass coverslips in 24-well tissue culture plates for 48 h. For use in flow cytometry, cells were cultured in 12-well tissue culture plates for 48 h. For analysis by Western blotting, cells were cultured in 6-well tissue culture plates for 48 h. Cells were prepared for doxycycline induction by plating and growth for 24 h followed by removal of the medium and addition of DMEM containing 5% FCS and 10 μg/ml of doxycycline (Sigma-Aldrich) for 24 h before further experiments were performed.

### Generation of expression and lentiviral vectors.

The plasmids pHβ-APr1-neo-*MUC1F*, pHβ-APr1-neo-*CT3*, and pHβ-APr1-neo-*CT33* were kindly provided by Michael A. Hollingsworth (University of Nebraska Medical Center). *MUC1F*, *CT3*, and *CT33* were excised with the restriction enzyme BamHI and ligated to the CMV promoter-containing vector pcDNA3.1(+), resulting in pcDNA3.1-*MUC1F* (pXL1), pcDNA3.1-*CT33* (pXL4), and pcDNA3.1-*CT3* (*ΔCT*) (pXL5). The vectors were sequenced with primers KS207aF (CTTGCCAGCCATAGCACCAAG), KS240F (CGCAAATGGGCGGTAGGCGTG), KS222R (GTGCTGGGATCTTCCAGAGAGG), and KS241R (TAGAAGGCACAGTCGAGG). A mutation was found in the SEA domain after aligning the *MUC1F* sequence with the human MUC1 cDNA sequence acquired from the Ensemble database and the Epi 1 MUC1 plasmid (a kind gift from John Hilkens). To correct the mutation, XagI and XbaI were used to cut Epi 1 and pXL1, then the fragment from Epi 1 was ligated to pXL1 to generate pXL2 and used as the *MUC1* vector in all studies. Synthetic DNA in which all the tyrosines in the MUC1 CT were substituted into phenylalanines was purchased from Thermo Fisher Scientific and ligated into the pXL2 backbone at the XagI and EcoRI sites to generate *MUC1-YF* (pXL25). To prepare lentiviral production vectors for doxycycline-inducible expression of the MUC1 constructs, the pInducer20 plasmid (Addgene number 44012) was adapted to contain an extended multiple cloning site containing NotI, NheI, HpaI, SalI, XhoI, and AscI restriction sites (GCGGCCGCTCAGGCTAGCGTAGTTAACTACGTCGACTCACTCGAGTTGGCGCGCC), and this plasmid was named pInducer20-extendedMCS (pKSU59). *MUC1-FL* was excised from pXL2 with the restriction enzyme XbaI and treated with Klenow fragment reagent, after which the product was digested with the restriction enzyme NheI and ligated to pKSU59 to generate pXL17 (pInducer-*MUC1*). pXL4, pXL5, and pXL25 were excised with the restriction enzymes NheI and XhoI and ligated into pKSU59 to generate pXL28 (pInducer-*MUC1-CT33*), pXL18 (pInducer-*MUC1-ΔCT*), and pXL27 (pInducer-*MUC1-YF*).

### Lentiviral production and generation of stable cell lines.

HeLa cells stably expressing MUC1-FL and MUC1-ΔCT were generated by lentiviral transduction. Subsequent FACS sorting was performed to enrich for MUC1-expressing cells. For lentiviral production, HEK293T cells were cultured to 90% confluence in 6-well tissue culture plates and transfected using Lipofectamine. For each well, 5.5 μl Lipofectamine 2000 (Life Technologies) was mixed with 150 μl Opti-MEM in an Eppendorf tube and incubated for 5 min at room temperature (RT). This mixture was transferred to another tube containing a mix of 1.5 μg lentiviral vector, 0.65 μg psPAX2, 0.35 μg pMD2.G, and 150 μl Opti-MEM. This mixture was incubated for 20 min at RT and subsequently slowly dropped onto the HEK293T cells in fresh DMEM/10% FCS. Cells were incubated for 6 h and replaced with fresh DMEM/10% FCS and grown for 48 h. Supernatants containing viral particles were harvested and filtered through a 0.2 μm filter and stored at −80°C or used directly to transduce target cells. For transduction, HeLa cells were plated and grown for 24 h to 90% confluence in 12-well tissue culture plates. The medium was removed and a mixture containing 250 μl of virus, 250 μl of DMEM/10% FCS, and 5 μl of Polybrene was added to the cells. Cells were incubated with the virus mixture for 8 to 10 h, after which the mixture was replaced with fresh DMEM/10% FCS. The next day, cells were washed with Dulbecco’s phosphate-buffered saline (DPBS) and detached with 300 μl of 0.05% trypsin-EDTA (Sigma), resuspended in DMEM/10% FCS, and transferred to 25 cm^2^ flasks. G-418 solution (04727878001, Roche) was added at a concentration of 500 μg/ml to select for transduced cells. Medium containing G-418 was exchanged every 2 days until no cells survived in the nontransduced control condition. To express the MUC1 constructs, cells were induced with 10 μg/ml of doxycycline for 24 h and stained with α-MUC1-ED antibody 139H2 (1:150, a kind gift from John Hilkens). The stable cell lines were subjected to FACS sorting to achieve a high percentage of MUC1-positive cells.

### Bacterial culture.

Escherichia coli DH5α strains expressing the invasin of Yersinia pseudotuberculosis (E. coli inv) (43) together with GFP or mCherry (30, 31) were routinely cultured at 37°C on Luria-Bertani (LB) agar plates or in 10 ml of LB broth with shaking at 160 rpm for 16 h with the appropriate antibiotics. E. coli DH5α and BL21 stains used for cloning and protein production were grown as above with the appropriate antibiotics.

### Infection assay.

Bacteria were grown for 16 h under standard conditions, and then diluted at 1:50 and subcultured for 2 h to reach mid-logarithmic phase, collected by centrifugation (8,000 rpm, 2 min), and resuspended in Dulbecco’s phosphate-buffered saline (DPBS, D8537, Sigma). Cell culture medium was replaced with DMEM without FCS prior to bacterial infection experiments. Bacteria were quickly added to cells at different multiplicities of infection (MOI) according to the assay used. When appropriate, 75 μM genistein (Sigma) or the solvent dimethyl sulfoxide (DMSO) (Sigma) were added to cells prior to bacterial infection and used throughout the assay. Infection was stopped by removing medium and rinsing the cells thoroughly with DPBS 3 times.

### Expression and purification of StcE and E447D.

The plasmids pET28b-StcE-Δ35-NHis and pET28b-StcE-E447D-Δ35-NHis were kindly provided by Natalie Strynadka (University of British Columbia). The construction of the two plasmids has been described previously (32). E. coli strain BL21 was transformed with the plasmids; single colonies were picked and grown at 37°C until an optical density of 0.6 to 0.8 was reached. Protein production was induced with 0.3 mM IPTG (isopropyl-β-D-thiogalactopyranoside) at 20°C overnight with shaking at 160 rpm. After induction, cultures were centrifuged at 4,000 × *g* for 15 min. Pellets were resuspended in 8 ml of binding buffer (NaH_2_PO_4_ buffered, pH 8.0, 10 mM imidazole). Lysozyme (1 mg/ml) was added and the suspension was incubated on ice for 30 min. Next, 1 μl of Pierce universal nuclease (88700, Thermo Fisher Scientific) was added and incubated at RT for 15 min. Pellets were lysed on ice by using a probe tip sonicator and (quick) freezing to −80°C for 30 min. Cell debris was removed by centrifugation at 4,000 × *g* for 15 min at 4°C. Lysates were applied to 1.5 ml Ni-NTA beads (88221, Thermo Fisher Scientific) for purification. The beads were rinsed once with 6 ml of MilliQ and twice with 6 ml of binding buffer. The lysates were then incubated with beads for 60 min on a shaker and followed by washing with 8 ml washing buffer (NaH_2_PO_4_ buffered, pH 8.0, 20 mM imidazole) for 4 times before eluting with elution buffer (NaH_2_PO_4_ buffered, pH 8.0, 250 mM imidazole). Pooled fractions for each enzyme were concentrated and exchanged with NaH_2_PO_4_ buffer using Spin-X UF 30 kDa MWCO filters (Sigma). Protein concentrations were determined using a BCA protein assay kit (23225, Thermo Fisher Scientific). Purified protein was stored at 4°C until use.

### StcE treatment of cells.

Five micrograms of StcE or E447D per 1 million epithelial cells in 1 ml of DMEM (10% FCS) was used to treat cells for 2 h at 37°C. After treatment, cells were washed with DPBS or DMEM (without FCS) when infection experiments followed.

### Confocal microscopy.

Cells were plated and grown for 24 h and followed by induction with 10 μg/ml doxycycline for 24 h where indicated. For infection experiments, bacteria were grown as described above and added at an MOI of 40. Cells were washed twice with DPBS and fixed with 4% cold paraformaldehyde in PBS (Affimetrix) for 20 min. For staining of the MUC1 ED, fixed cells on coverslips were permeabilized in binding buffer containing 0.1% saponin (Sigma) and 0.2% BSA (Sigma) in DPBS for 30 min and washed two times with DPBS and incubated onto 50-μl drops containing 139H2 antibody diluted 1:150 in binding buffer on parafilm for 1 h at RT. For staining of the MUC1 SEA domain, cells on coverslips were permeabilized in DPBS containing 0.2% Triton X-100 (Merck) for 10 min. Then cells were blocked with 1% BSA and 22.52 mg/ml glycine in PBST (DPBS + 0.1% Tween 20 [Sigma]) for 30 min and washed three times with DPBS. Cells were incubated onto 100-μl drops of the α-MUC1-SEA antibody 232A1 (a kind gift from John Hilkens) diluted 1:100 with 1% BSA in PBST on parafilm overnight at 4°C. After removing the primary antibody, 4 washing steps were performed. The coverslips were further incubated with the secondary antibodies Alexa Fluor 488-conjugated goat α-mouse IgG (1:200; A11029, Thermo Fisher) and DAPI at 2 μg/ml (D21490, Invitrogen) for 1 h. Coverslips were washed 3 times with DPBS, washed a final time with MilliQ, dried, embedded in Prolong diamond mounting solution (Thermo Fisher), and allowed to solidify. A Leica SPE-II confocal microscope (Leica Microsystems, Wetzlar, Germany) was used to acquire single plane images with a 63× objective (NA 1.3, HCX PLANAPO oil) controlled by Leica LAS AF software with default factory settings. A quad band dichroic was used, allowing diode laser wavelengths 405, 488, 561 nm lines to pass and fluorescent signal to enter the prism to sequentially detect DAPI, Alexa Fluor 488, and mCherry. Images stacks were collected on a NIKON Ti-E stand connected to an A1Rs confocal microscope (NIKON instruments Inc., Tokyo, Japan) using a 100× oil immersion objective (NIKON TIRF Apochromat NA1.49) in bidirectional resonance mode (average 16). Sequential laser illumination through a quadband dichroic (405, 488, 561, and 647) was used in combination with emission filters 460/60, 515/30, 595/50, and 700/75 to detect DAPI, Alexa Fluor 488, mCherry, and Alexa Fluor 647, respectively. Acquired images were processed in NIS elements 5.2 (NIKON). Denoising was executed using the ai package and 3D deconvolution was performed by the Blind method with point spread function spherical aberration correction set for the acquisition depth. Maximum intensity projections are shown, as well as representative slices from the image series. Linear intensity adjustment was optimized for each label combination except for the DAPI signal that required variable gamma adjustment to highlight nuclear contours. Final outlining of the figures was performed in Adobe Illustrator (Adobe Inc., San Jose, USA).

### Flow cytometry.

Cells were plated and grown for 24 h, induced with 10 μg/ml doxycycline for 24 h, and then washed three times with DMEM (without FCS). For infection experiments, bacteria were grown as described above and added at an MOI of 20. Adherent cells were detached by incubating them with 200 μl of 0.05% trypsin-EDTA at 37°C in 5% CO_2_ for 5 min. Cells were taken up with cold FACS buffer (2% BSA in DPBS) and centrifuged at 1,500 rpm for 5 min. Cells were fixed with 4% cold paraformaldehyde in PBS for 10 min. α-MUC1-ED antibody 139H2 (1:100), Alexa Fluor 568-conjugated goat α-mouse IgG secondary antibody (1:50; A11031, Thermo Fisher), or phycoerythrin (PE) goat α-mouse IgG secondary antibody (1:1,600; 1031-09, SouthernBiotech) were diluted in FACS buffer and incubated with cells on ice for 30 min in the dark. Data were collected on a Beckman Coulter CytoFLEX and analyzed with FlowJo software (TriStar).

### Western blotting.

Cells were washed twice with cold DPBS and collected with a scraper. For StcE-treated cells, 0.5 M EDTA was added to quench the enzyme before collection of the cells. The cell suspension was centrifuged at 1,500 rpm for 5 min at 4°C. Cell pellets were resuspended with 150 μl cold NP-40 lysis buffer (150 mM Nacl, 1.0% Nonidet P-40, and 50 mM Tris-HCl [pH 7.4]). Three times concentrated Laemmli sample buffer was added to the lysate, which was then boiled for 5 min at 100°C. For detection of the MUC1 ED, 5% mucin gel and a boric acid-Tris system were used as described previously (12). α-MUC1-ED antibody 214D4 (CD227, Nordic MUBio) was used to detect MUC1 and used at a dilution of 1:150 in TSMT buffer as described previously (12). For detection of the CT of mucins, α-MUC1-CT antibody F33 (1:1,000, a kind gift from John Hilkens) was used. Actin was detected using α-actin antibody (1:5,000; bs-0061R, Bioss). Secondary antibodies used were α-mouse IgG secondary antibody (1:5,000; A2304, Sigma) and α-rabbit IgG (1: 5,000; A4914, Sigma). Blots were developed with the Clarity Western ECL kit (Bio-Rad) and imaged in a Gel-Doc system (Bio-Rad).
