# Supplementary figures and images for: The Transmembrane Mucin MUC1 Facilitates β1-Integrin-Mediated Bacterial Invasion
Source: mBio. 2021 Apr 6;12(2):e03491-20. doi: 10.1128/mBio.03491-20 (PMC8092303; doi:10.1128/mBio.03491-20)

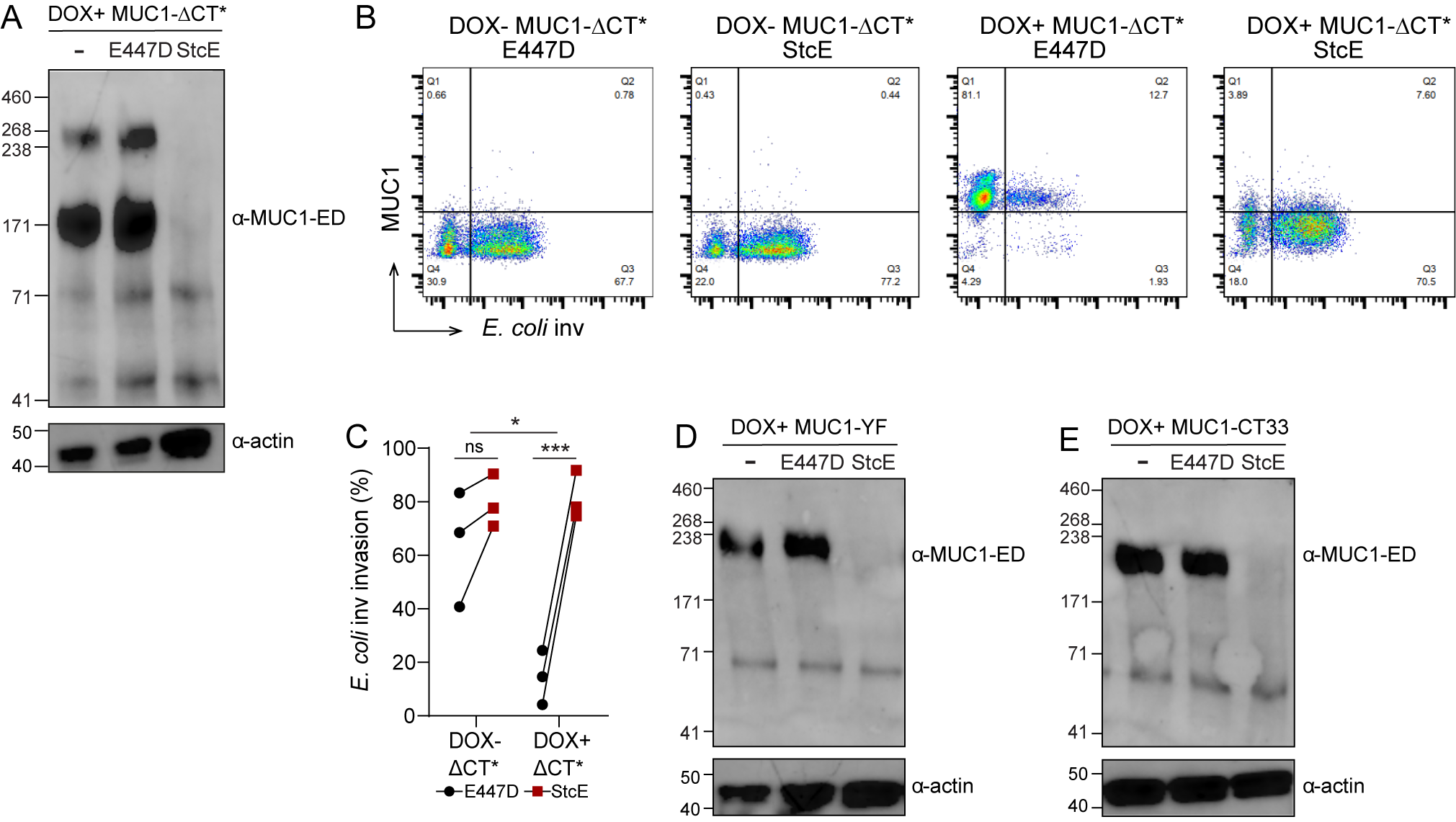

Supplement: FIG S3 [file mBio.03491-20-sf003.tif]

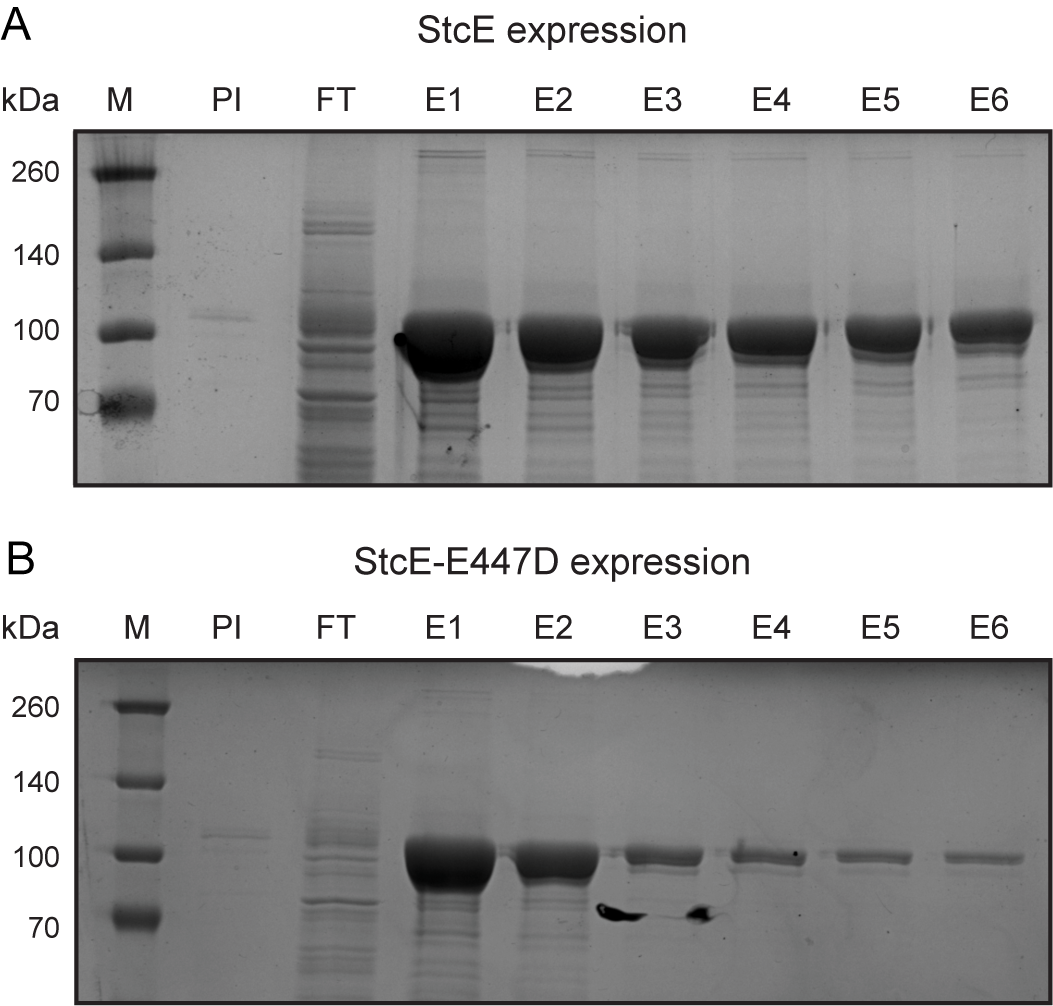

Supplement: FIG S1 [file mBio.03491-20-sf001.tif]

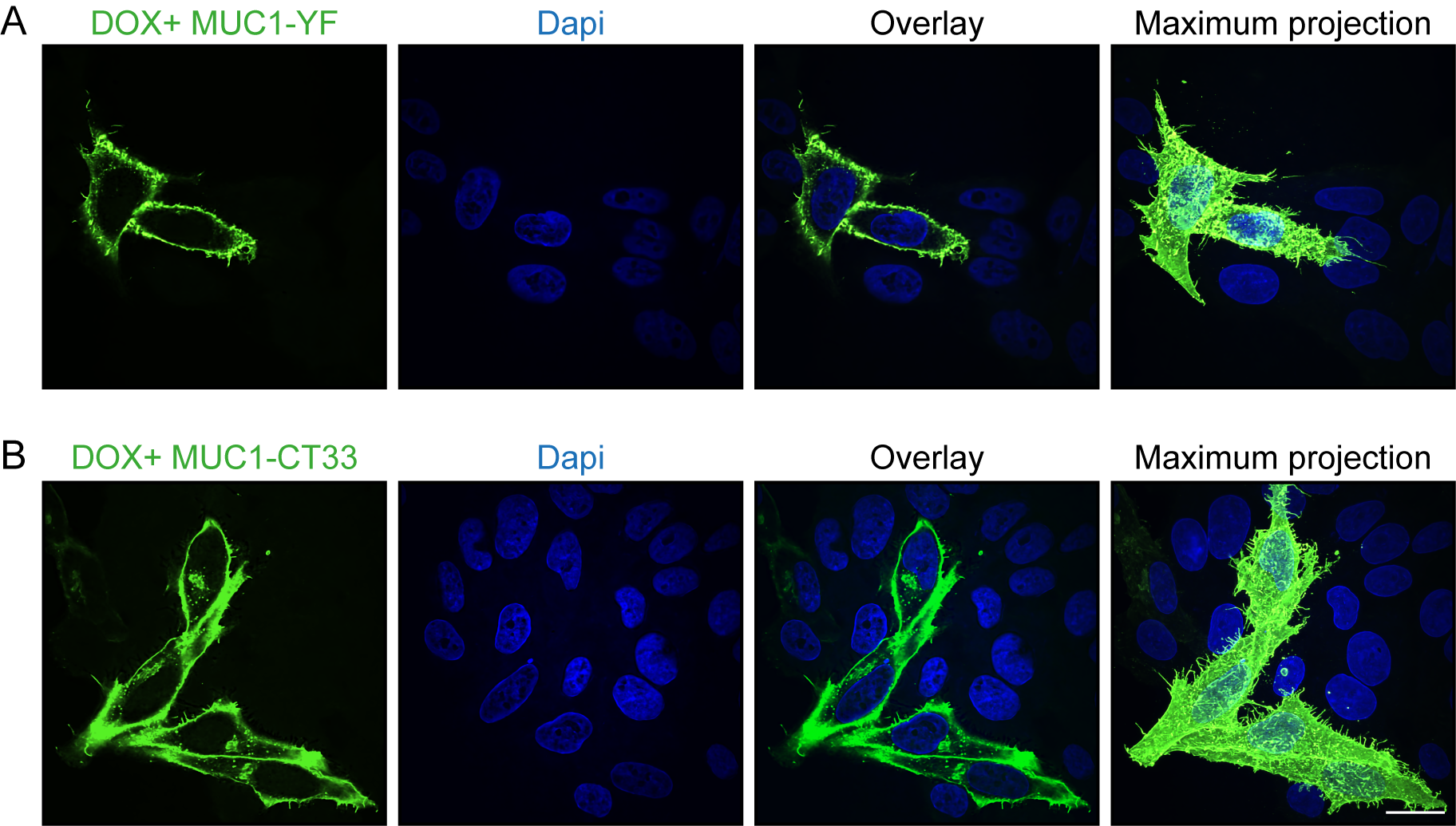

Supplement: FIG S2 [file mBio.03491-20-sf002.tif]
